# Supplementary material for: Sensing of HIV-1 by TLR8 activates human T cells and reverses latency
Source: Nat Commun. 2020 Jan 9;11:147. doi: 10.1038/s41467-019-13837-4 (PMC6952430; doi:10.1038/s41467-019-13837-4)
Supplement: Supplementary file 1 — Supplementary Information [file 41467_2019_13837_MOESM1_ESM.pdf]

Supplementary Information

Sensing of HIV-1 by TLR8 activates human T cells and reverses latency

H. Z. Meås, M. Haug et al.

This PDF file includes:

Supplementary Figures 1-7

Supplementary Tables 1-2

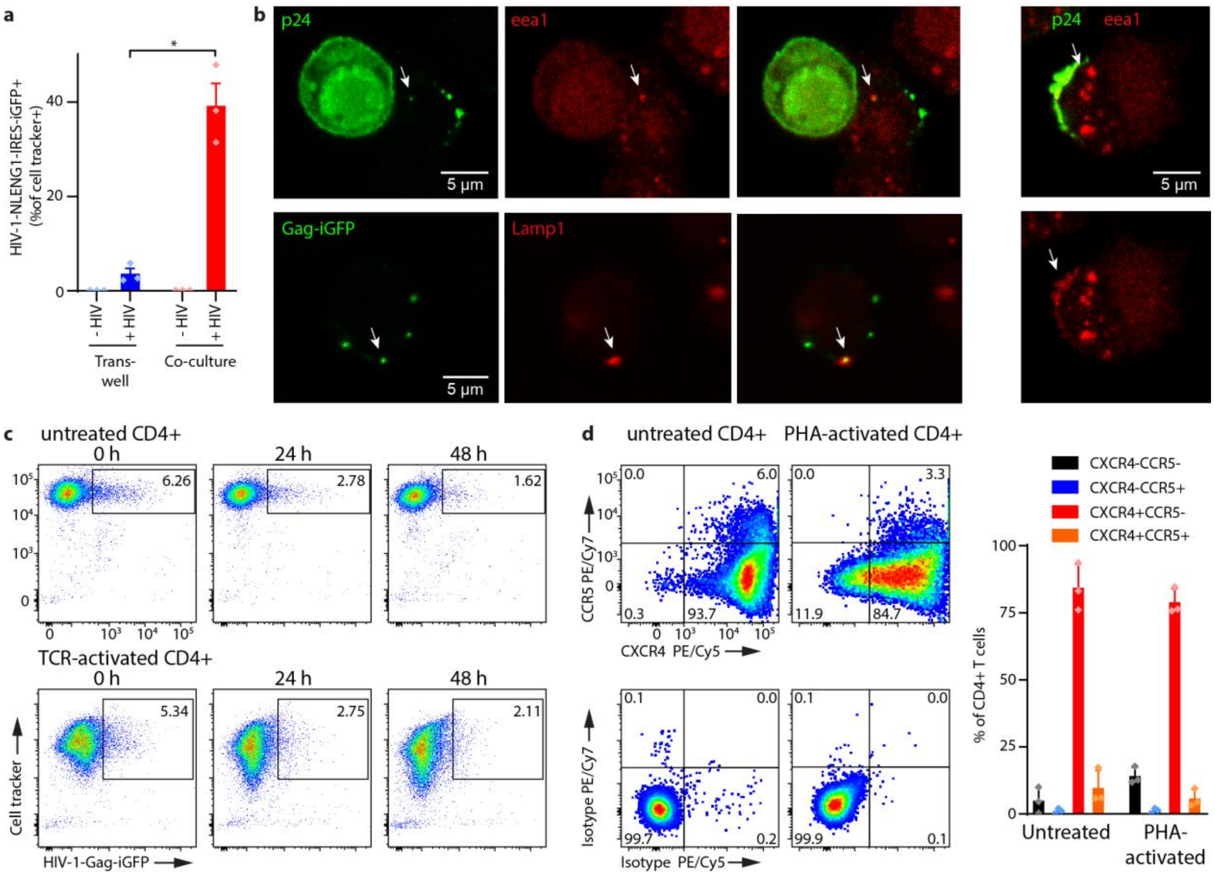

Supplementary Fig. 1. Characterization of intracellular HIV compartments and frequencies of CD4+ T cells expressing CXCR4/CCR5.

a SupT1 were infected with NLENG1-IRES-eGFP virus for 48h prior to coculture with TCR-activated CD4+ (stained with CellTracker Red) together (co-culture) or separated (trans-well) by 0.4 µm trans-well porous membrane barrier for 4 days. Frequencies of T cells (cell tracker red) expressing eGFP were quantified by flow cytometry, data represent means + SEM from three independent experiments. Statistical significance was determined by two-sided paired t-test on log transformed data, *$P < 0.05$. **b** Examples of EEA1 and LAMP1-positive compartments in CD4+ T cells containing HIV puncta (stained by anti-p24) after co-culture with HIV-1-expressing HEK293 cells. **c** Untreated or TCR-activated CD4+ T cells were co-cultured with HeLa cells expressing X4-tropic HIV-1-Gag-iGFP in the presence of the CXCR4 inhibitor AMD3100. Frequencies of T cells (cell tracker red) harboring HIV-1 (iGFP) were quantified by flow cytometry 0, 24 and 48h post infection/co-culture. Representative dot plot examples from a representative donor corresponding to quantification in main Fig. 1c. **d** Dot plot examples (left) and quantification (right) showing the frequency of untreated and PHA activated CD4+ T cells expressing CCR5 and CXCR4. Bottom dot plots show isotype controls, bars represent mean + SEM from three independent experiments. Source data are provided as a Source Data File.

a

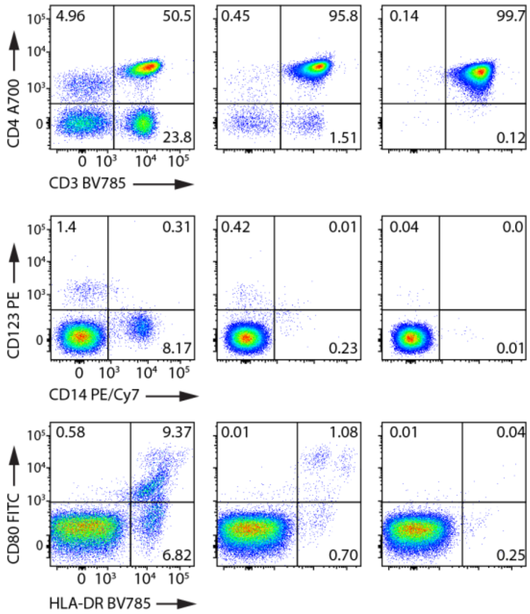

b

	PBMCs	"Negative" isolation	"Positive" isolation
	Mean *(SD)*	Mean *(SD)*	Mean *(SD)*
CD3+CD4+	50,86 *(7,56)*	95,75 *(1,17)*	99,55 *(0,19)*
CD3+CD8+	18,44 *(6,49)*	0,34 *(0,57)*	0,61 *(0,43)*
CD14+	6,87 *(5,07)*	0,18 *(0,07)*	0,27 *(0,09)*
CD19+	3,37 *(3,66)*	0,05 *(0,10)*	0,10 *(0,07)*
CD16+	13,46 *(6,35)*	1,37 *(0,54)*	0,13 *(0,11)*
CD303+	0,27 *(0,20)*	0,14 *(0,12)*	0,06 *(0,06)*
CD123+	1,88 *(0,38)*	0,24 *(0,26)*	0,53 *(1,00)*
HLA-DR+	12,74 *(5,69)*	0,95 *(0,68)*	0,47 *(0,29)*
CD80+	7,14 *(8,36)*	0,62 *(0,51)*	0,47 *(0,58)*

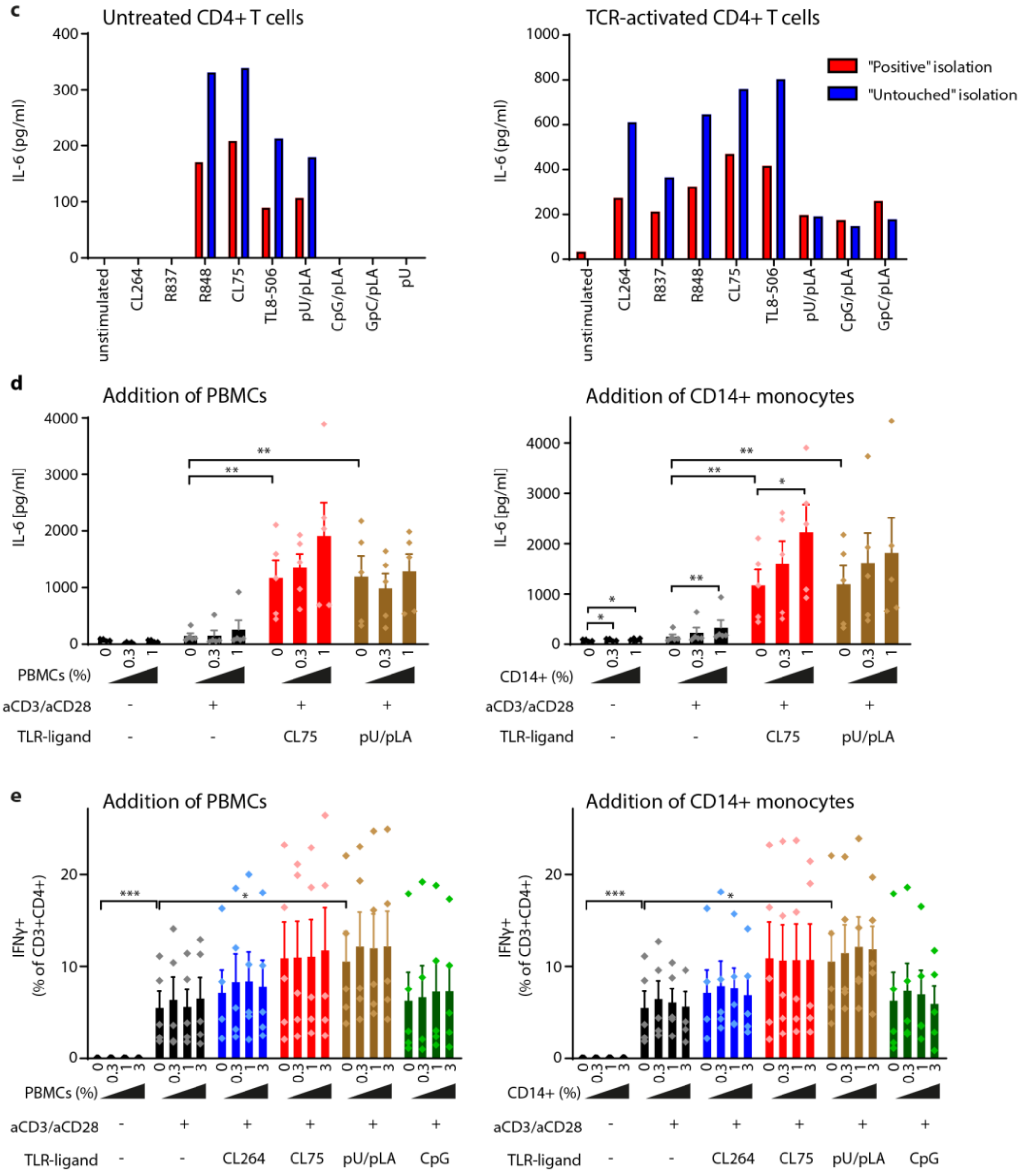

Supplementary Fig. 2. Impact of T cell purity on TLR ligand induced IL-6 production.

a, b Flow cytometric analysis of cell populations in PBMCs and CD4+ T cells isolated from PBMCs by depleting non-T cells (negative isolation) or via positive isolation using antibody-coated beads. **a** Representative example of CD4+ T cell purity in the different isolations. **b** Cell population frequencies determined from expression of select surface markers. Values represent mean +/- SD from 5 independent experiments (4 for PBMCs). **c** Comparison of TLR-induced IL-6 production from CD4+ T cells negatively ("untouched", purity > 95%) or positively (purity > 99 %) isolated from one donor. Untreated and TCR-activated CD4+ T cells from both populations were stimulated with 5 µg/ml ligands to TLR7 (CL264, R837), TLR7/8 (R848), TLR8 (CL75, TL8-506, pU/pLA) or TLR9 (CpG, GpC, 5 µM) for 24h. IL-6 in supernatants was analyzed by ELISA. Data are from one experiment. **d** Purified CD4+ T cells were activated by TCR +/- TLR-ligand stimulation in the presence of 0, 0.3 or 1 % of PBMCs depleted of CD4+ cells (left) or CD14+ monocytes (right). IL-6 secretion was measured by ELISA from supernatants 24 h post stimulation. Data represents means + SEM from five independent experiments. **e** Purified CD4+ T cells were combined with increasing doses (0-3%) of PBMCs depleted of CD4+ cells (left) or CD14+ monocytes (right) and activated by TCR +/- TLR-ligand stimulation for 48h before intracellular IFN-γ production from CD4+ T cells was assessed by flow cytometry, data represent means + SEM from five independent experiments. Statistical significance in **d** and **e** was determined from log-transformed data by two-way ANOVA with Dunnett´s post-test; Significance levels: *$p < 0.05$; **$p < 0.01$; ***$p < 0.001$. Source data are provided as a Source Data File.

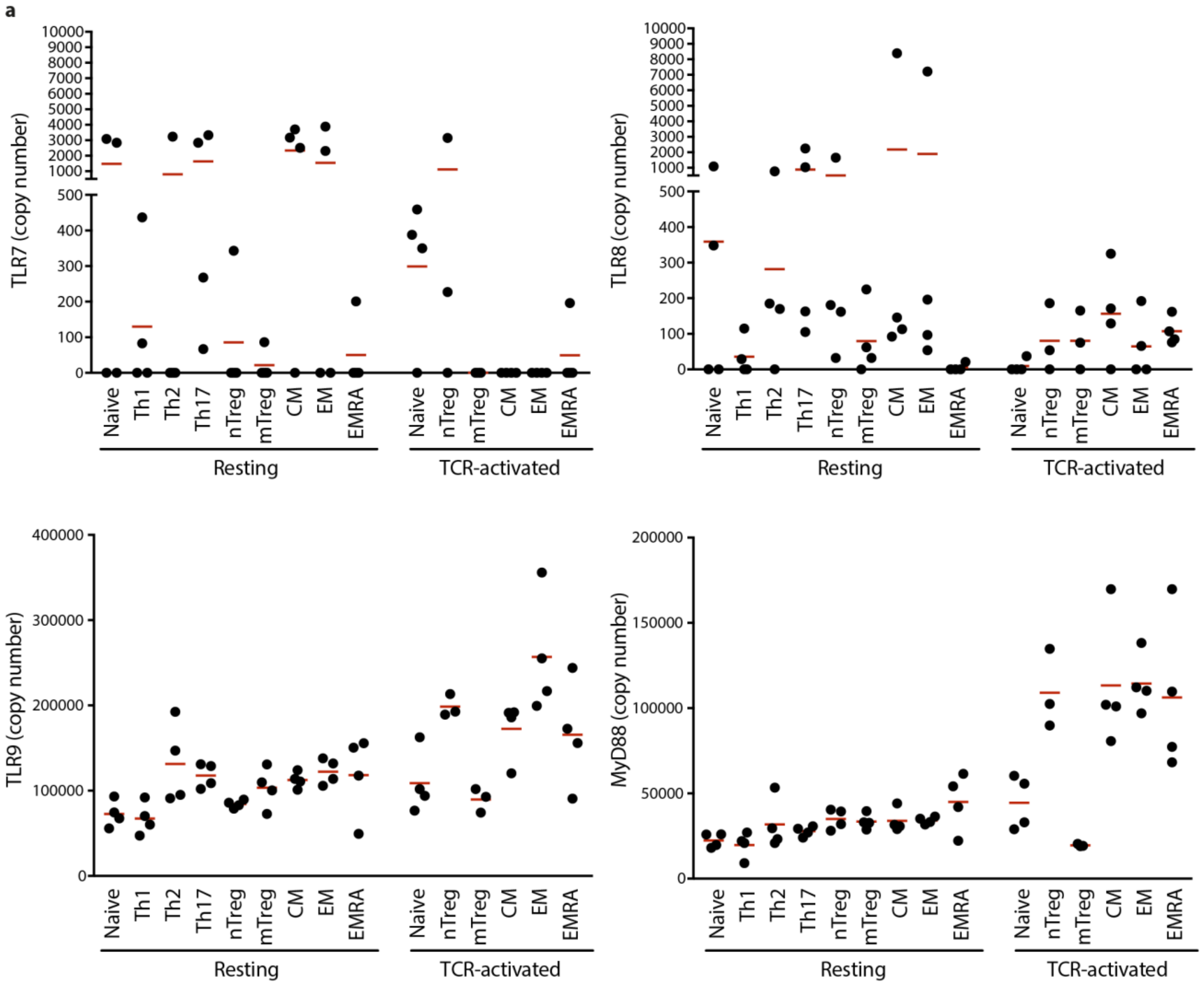

Supplementary Fig. 3. Resting and TCR-activated CD4+ T cells express TLR7/8/9 and MyD88. Protein copy numbers of TLR7/8/9 and MyD88 in resting and TCR-activated subsets of CD4+ T cells. Plots were generated from quantitative mass spectrometry-based proteomics data on FACS sorted CD4+ T cell subsets obtained from Rieckmann *et al.*, doi: 10.1038/ni.3693. Mean values are indicated with red lines. Th = T helper; nTreg, mTreg = naïve or memory regulatory T cells; CM, EM = central or effector memory T cells; EMRA = effector memory RA+ T cells.

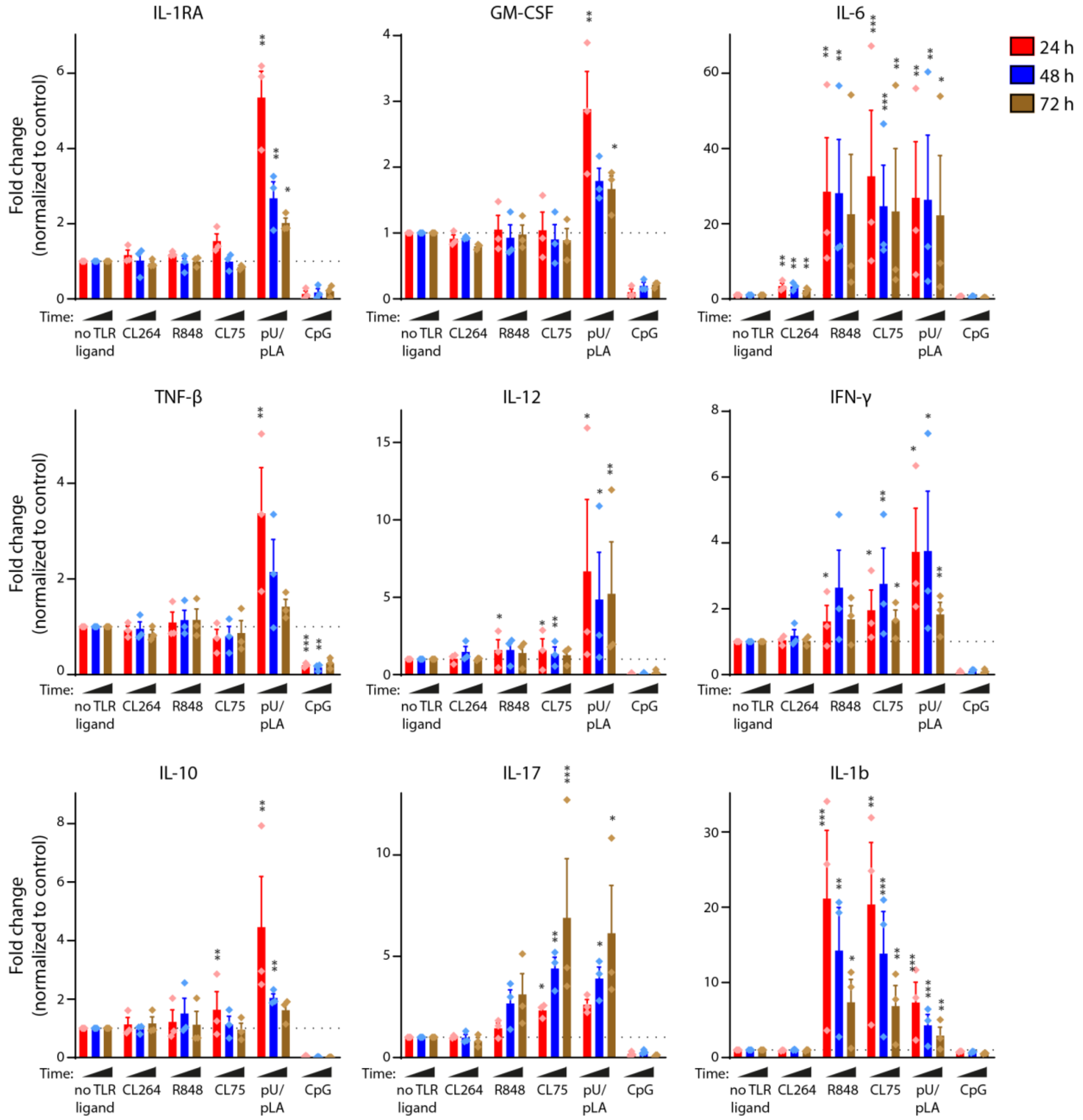

Supplementary Fig. 4. Cytokine responses to TLR 7, 8 and 9 ligands in CD4+ T cells.

CD4+ T cells were TCR activation in combination with 5 µg/ml CL264 (TLR7), R848 (TLR7/8), CL75, pU/pLA (TLR8) or CpG (TLR9, 5 µM) for the times indicated. Cytokine levels in cell supernatants were analyzed using multiplex ELISA. Graphs represent mean values + SEM from 3 independent experiments normalized to TCR-activated CD4+ T cells activated without addition of TLR-ligands. Statistical significance was determined from log-transformed raw-data by two-way ANOVA followed by Dunnett´s post-test. Significance levels from raw-data are indicated in the graphs for data with a more than 1.5-fold relative increase/decrease compared to TCR-activated cells; $^{*}p < 0.05$; $^{**}p < 0.01$, $^{***}p < 0.001$. Source data are provided as a Source Data File.

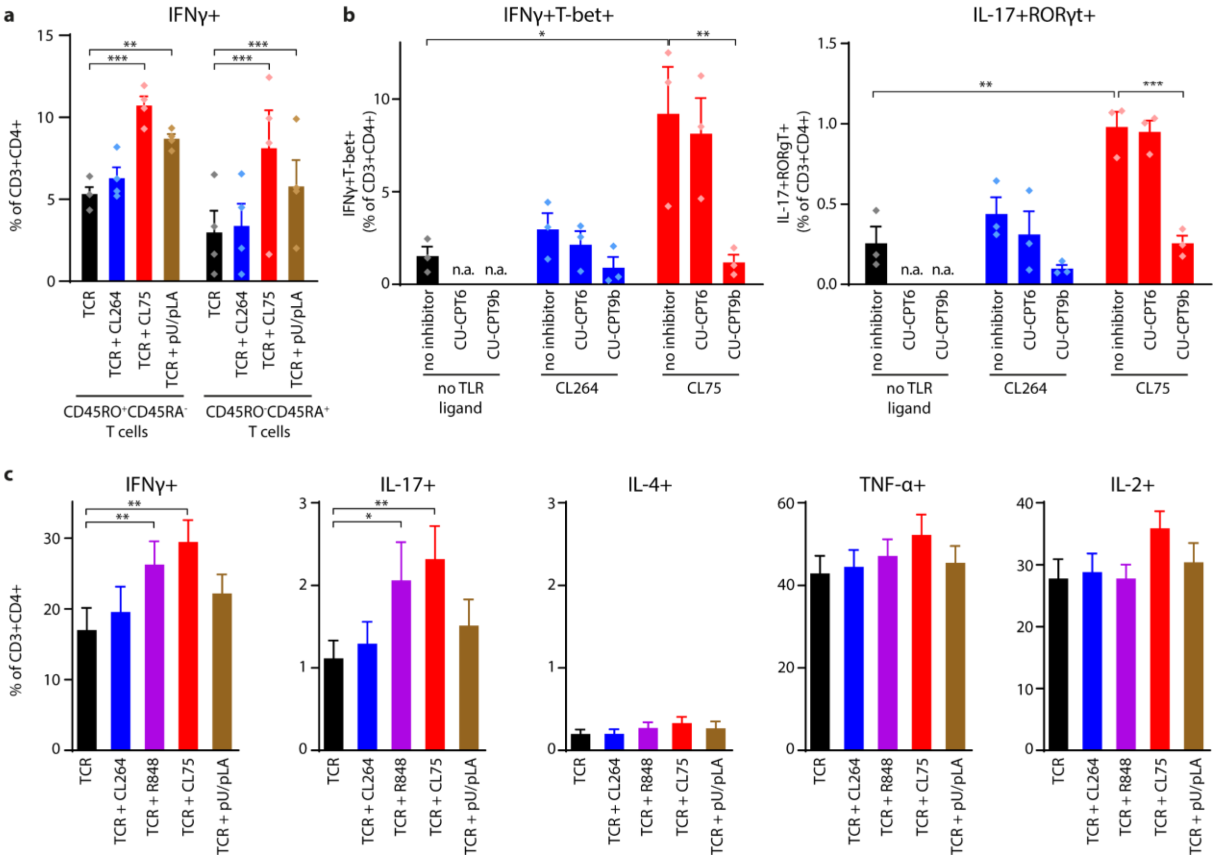

Supplementary Fig. 5. Effect of TLR8 stimulation on T helper cell lineage commitment.
a Primary CD4+ T cells were TCR-activated in the presence or absence of 5 µg/ml CL264, CL75 or pU/pLA for 48h. Production of IFN-γ from CD4+ T cells with a memory (CD45RO+CD45RA-) or naïve (CD45RO-CD45RA+) phenotype was analyzed by intracellular flow cytometry. Bars represent mean + SEM from four independent experiments. **b** Primary CD4+ T cells were TCR-activated in the presence or absence of 5 µg/ml CL264 or CL75 and 5 µM TLR8 inhibitor (CU-CPT9b) or control (CU-CPT6) for 72h. The fraction of T cells expressing the lineage-indicating transcription factors T-bet or RORγt and producing IFN-γ or IL-17 was analyzed by intracellular flow cytometry. Bars represent mean + SEM from three independent experiments. **c** CD4+ T cells were differentiated for 8 days into CD4+ effector T cells by TCR-activation in the presence or absence of 5 µg/ml CL264 (TLR7), R848 (TLR7/8), CL75 or pU/pLA (TLR8). On day 8, cells were re-stimulated with PMA/ionomycin in the presence of protein-transport inhibitor for 6h and analyzed by intracellular flow cytometry for cytokine production. Percentages of CD3+CD4+ T cells producing the indicated cytokines are shown. Bars represent mean + SEM from twelve independent experiments. Statistical significance was determined from log-transformed data by one-way ANOVA on memory and naïve datasets in **a** and data in **c**, by two-way ANOVA following Dunnett´s post-test for data in **b**; **p* < 0.05; ***p* < 0.01, ****p* < 0.001. Source data are provided as a Source Data File.

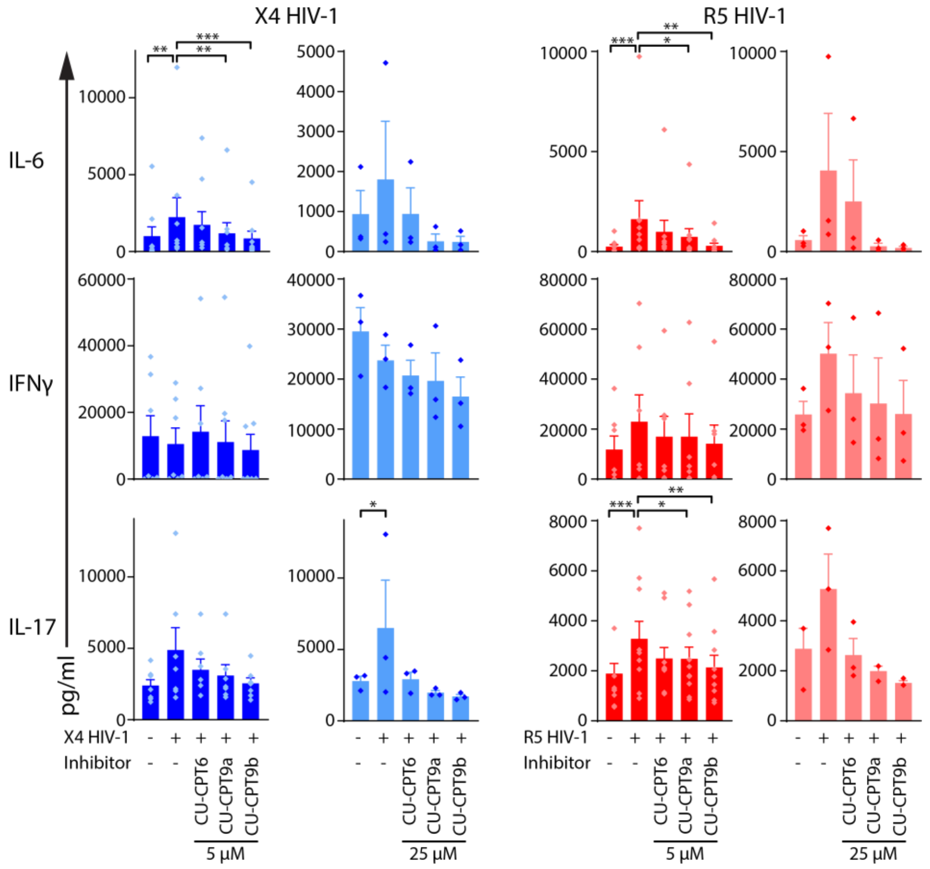

Supplementary Fig. 6. Cytokine secretion from CD4+T cells with endo-lysosomal HIV-1 infection. Primary CD4+ T cells were pre-treated with CXCR4 inhibitor AMD3100 or CCR5 inhibitor Maraviroc for 24h, and TLR8-specific inhibitors CU-CPT9a, CU-CPT9b or the negative control compound CU-CPT6 for 2h, prior to concomitant TCR activation and co-culture with HEK293 cells infected with HIV-1-Gag-iGFP (X4 HIV-1) or HIV-Gag-iGFP JRFL (R5 HIV-1). Cytokines were analyzed in the supernatant 24h post infection by multiplex ELISA. Bars represent means + SEM from 7-10 independent experiments with 5 µM inhibitor and 3 independent experiments with 25 µM inhibitor. Statistical significance was determined by repeated measures one-way ANOVA with Dunnett´s post-test on log-transformed data; *$p < 0.05$; **$p < 0.01$; ***$p < 0.001$. Source data are provided as a Source Data File.

a Gating strategy HIV-1 infected CD4+ T cells (Figure 1c, Figure 7 and Supplementary Figure 1c)

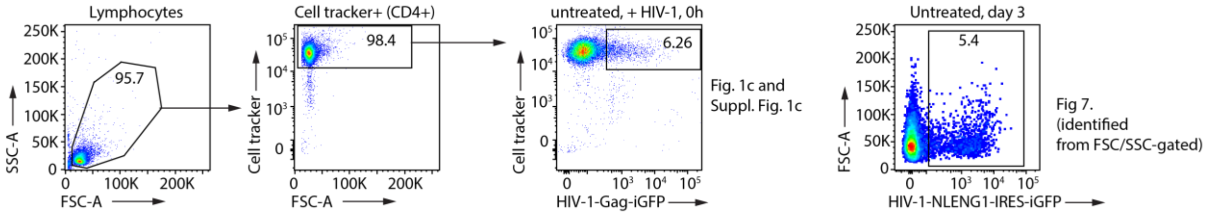

b Gating strategy HIV-1 infected CD4+ T cells by imaging flow cytometry (Figure 1d)

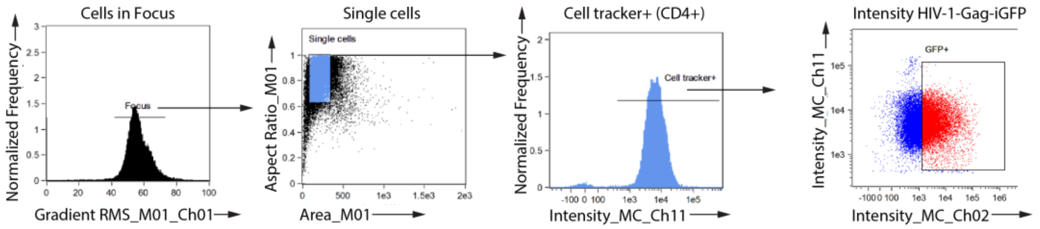

c Gating strategy CD4+ T cell activation markers (Figure 3b)

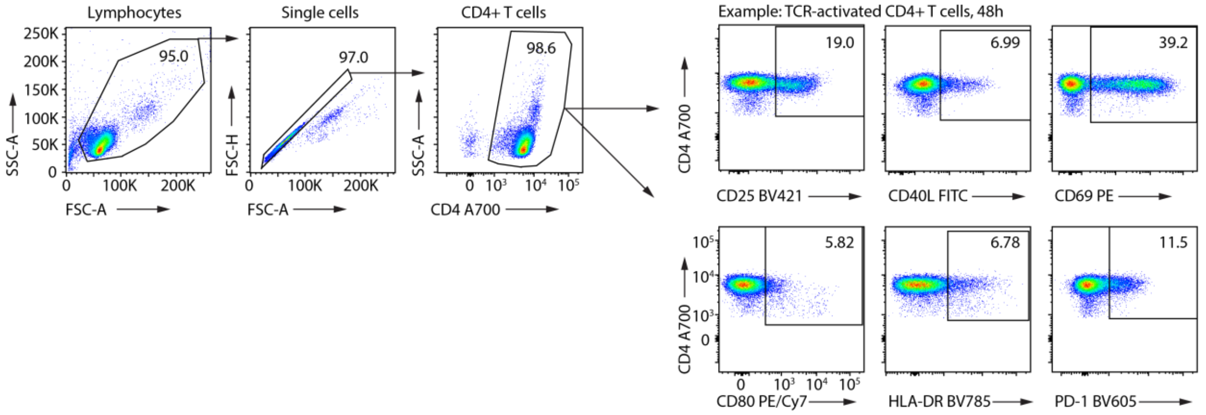

d Gating strategy phospho-flow cytometry (Figure 4)

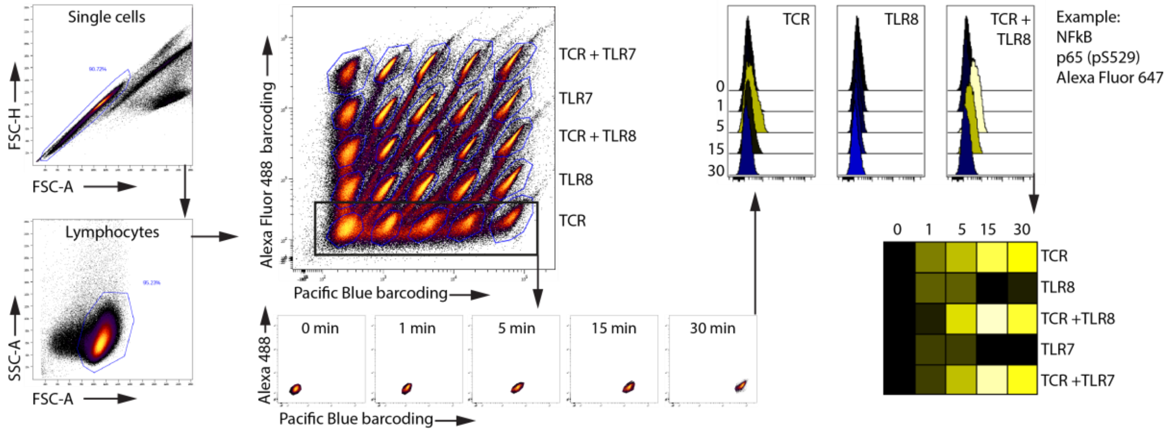

e Gating strategy cytokine-producing CD4+ T cells (Figures 5b+c+d, Supplementary Figures 2e, 5a+c)

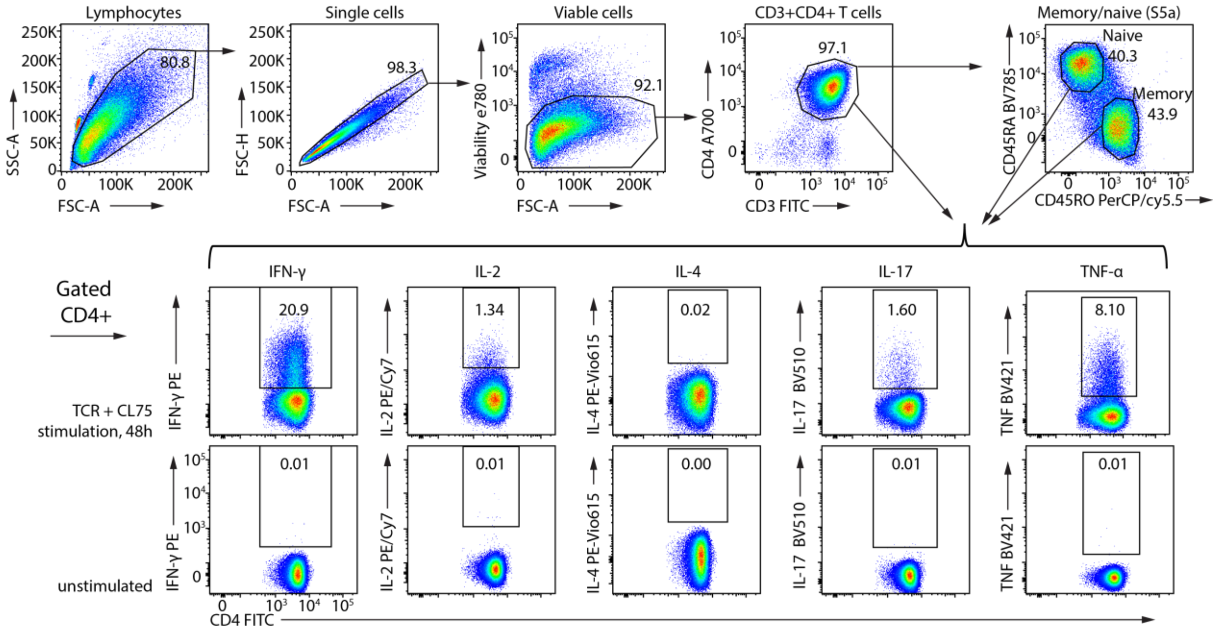

f Gating strategy transcription factors and cytokine-production (Supplementary Figure 5b)

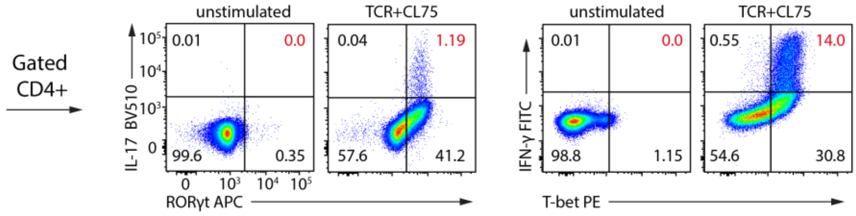

g Gating strategy transwell-experiment (Supplementary Figure 1a)

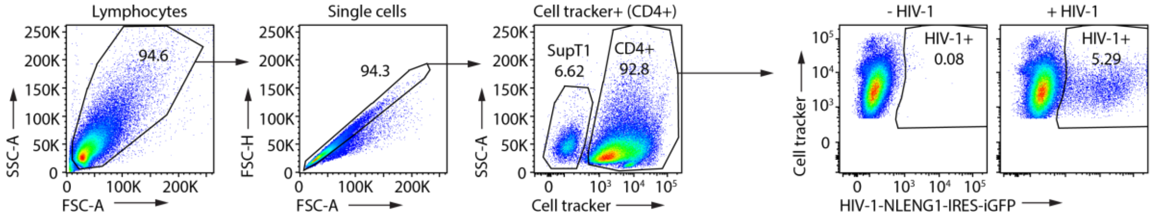

h Gating strategy CXCR4/CCR5 expression on CD4+ T cells (Supplementary Figure 1d)

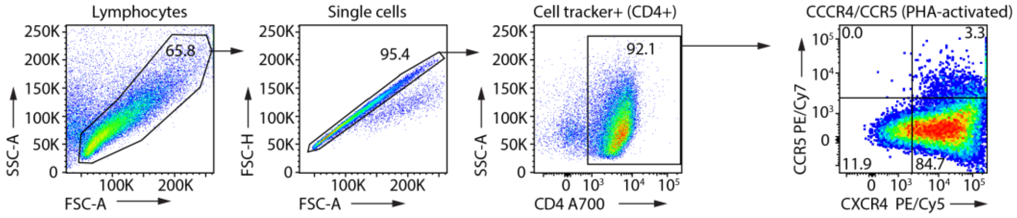

i Gating strategy CD4+ T cell purity analysis (Supplementary Figure 2a+b)

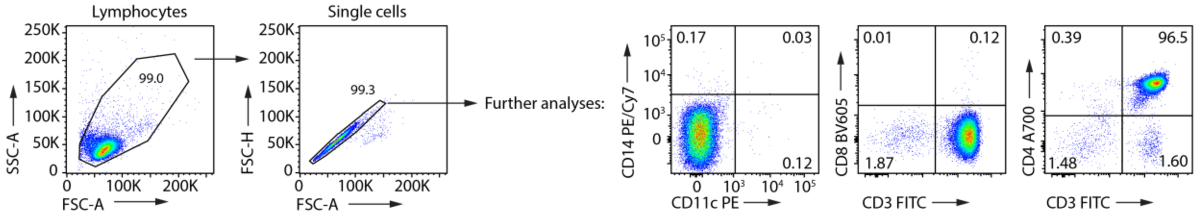

Supplementary Fig. 7. Gating strategies.

a Gating strategy to identify HIV-1 infected CD4+ T cells in Figure 1c, Supplementary Figure 1c and Figure 7. **b** Gating strategy to identify HIV-1 infected CD4+ T cells by imaging flow cytometry in Figure 1d. **c** Gating strategy for analysis of CD4+ T cell activation marker expression in Figure 3b. **d** Gating strategy for phosphorylation analysis of signaling proteins in Figure 4. **e** Gating strategy for analysis of effector cytokine production from CD4+ T cells and memory and naïve subsets in Figures 5b, c and d, Supplementary Figure 2e and Supplementary Figures 5a and c. **f** Gating strategy for analysis of transcription factor expression and effector cytokine production from CD4+ T cells in Supplementary Figure 5b. **g** Gating strategy for analysis of HIV-1 positive CD4+ T cells in transwell-experiment in Supplementary Figure 1a. **h** Gating strategy for analysis of CXCR4 and CCR5 expression on CD4+ T cells in Supplementary Figure 1d. **i** Gating strategy for CD4+ T cell purity analysis shown in Supplementary Figure 2a and b. All gates for cytokine production, protein phosphorylation, surface marker and transcription factor expression as well as HIV-1 infection were defined from unstimulated or uninfected samples.

Supplementary Table 1: HIV patients characteristics.

Patient ID	Sex	Age	Viral load copies/ml	CD4+ cells/μl	Treatment regimen
1	F	69	< 20	790	Efavirenz / Emtricitabine / Tenofovir
2	M	42	undetectable	790	Efavirenz / Emtricitabine / Tenofovir
3	M	45	undetectable	280	Efavirenz / Emtricitabine / Tenofovir
4	M	35	undetectable	710	Rilpivirine / Emtricitabine / Tenofovir
5	M	27	undetectable	720	Rilpivirine / Emtricitabine / Tenofovir
6	F	37	undetectable	1330	Lopinavir + Ritonavir / Emtricitabine / Tenofovir
7	M	30	119	190	Efavirenz / Emtricitabine / Tenofovir
8	M	57	undetectable	470	Atazanavir / Ritonavir, Emtricitabine / Tenofovir
9	M	39	< 20	460	Darunavir / Cobicistat, Emtricitabine / Tenofovir

Supplementary Table 2.

Antibodies for confocal microscopy (CM), Western Blot (WB), flow cytometry (FC), cell stimulation (S) and cell depletion (D).

Epitope	Conjugate	Clone	Supplier	Catalog number	Dilution
EEA1	unconjugated	polyclonal	Santa Cruz	sc-33585	1:100 (CM)
HIV1 p24	unconjugated	39/5.4A	abcam	ab9071	1:100 (CM)
LAMP1	unconjugated	H4A3	Santa Cruz	sc-20011	1:100 (CM)
LAMP1	unconjugated	polyclonal	abcam	ab24170	1:100 (CM)
Goat anti-mouse (LAMP1 Santa Cruz, p24, GPF)	Alexa Fluor 555	polyclonal	Thermofisher	A-21422	2µg/ml (CM)
Goat anti-rabbit (LAMP1 abcam, EEA1)	Alexa Fluor 405	polyclonal	Thermofisher	A-31556	2µg/ml (CM)
Goat anti-rabbit	HRP	polyclonal	Dako	P0448	1:(2000 x F) (WB)
TLR8	unconjugated	D3Z6J	Cell Signaling Technology	11886	1:1000 (WB)
COX4	unconjugated	SP2	abcam	ab33985	1:1000 (WB)
Akt (pS473)	Alexa Fluor 647	D9E	Cell Signaling Technology	4075	1:25 (FC)
CCR5 (CD195)	PE/Cy7	J418F1	BioLegend	359107	1:100 (FC)
CD11c	PE	3.9	eBioscience	12-0116-42	1:50 (FC)
CD123	PE	AC145	Miltenyi Biotec	130-113-326	1:50 (FC)
CD14	PE/Cy7	61D3	eBioscience	25-0149-42	1:50 (FC)
CD16	FITC	NKP15	BD Biosciences	347523	1:10 (FC)
CD19	PE	HIB19	eBioscience	12-0199-42	1:50 (FC)
CD25	Brilliant Violet 510	M-A251	BioLegend	356120	1:50 (FC)
CD25	PE	4E3	Miltenyi Biotec	130-113-282	1:25 (FC)
CD3	FITC	OKT3	eBioscience	11-0037-42	1:100 (FC)
CD3	Brilliant Violet 785	OKT3	BioLegend	317330	1:100 (FC)
CD303	APC	AC144	Miltenyi Biotec	130-113-190	1:50 (FC)
CD3ζ (CD247) (pY142)	Alexa Fluor 647	K25-407.69	BD Biosciences	558489	1:10 (FC)
CD4	Alexa Fluor 700	OKT4	eBioscience	56-0048-82	1:100 (FC)
CD40L	FITC	24-31	BioLegend	310804	1:100 (FC)
CD45RA	Brilliant Violet 785	HI-100	BioLegend	304140	1:100 (FC)
CD45RO	PerCP/Cy5.5	UCHL1	BioLegend	304222	1:100 (FC)
CD69	PE	FN50	BioLegend	310906	1:100 (FC)
CD8	Brilliant Violet 605	M5E2	BioLegend	301814	1:100 (FC)
CD80	PE/Cy7	2D10	BioLegend	305218	1:100 (FC)
CD80	FITC	L307	BD Biosciences	557226	1:10 (FC)
CXCR4 (CD184)	PE/Cy5	12G5	BioLegend	306507	1:100 (FC)
HLA-DR	Brilliant Violet 785	L243	BioLegend	307642	1:100 (FC)
IFN-γ	PE	4S.B3	eBioscience	12-7319-42	1:50 (FC)

IFN-γ	FITC	REA600	Miltenyi Biotec	130-113-497	1:25 (FC)
IkB alpha (pS32/pS36)	eFluor 660	RILYB3R	eBioscience	50-9035-42	1:10 (FC)
IL-17	Brilliant Violet 510	BL168	BioLegend	512330	1:50 (FC)
IL-2	PE/Cy7	MQ1-17H12	eBioscience	25-7029-42	1:50 (FC)
IL-4	PE-Vio615	7A3-3	Miltenyi Biotec	130-107-144	1:25 (FC)
NF-κB p65 (pS529)	Alexa Fluor 647	K10-895.12.50	BD Biosciences	558422	1:33 (FC)
NF-κB p65 (pS536)	Alexa Fluor 647	93H1	Cell Signaling Technology	4887	1:100 (FC)
p38 MAPK (pT180/pY182)	Alexa Fluor 647	28B10	Cell Signaling Technology	4552	1:50 (FC)
p44/42 MAPK (Erk1/2, pT202/pY204)	Alexa Fluor 647	E10	Cell Signaling Technology	4375	1:100 (FC)
PD-1	Brilliant Violet 605	EH12.2H7	BioLegend	329924	1:100 (FC)
RORγt	APC	AFKJS-9	eBioscience	17-6988-82	1:50 (FC)
S6 (pS235/ pS236)	Alexa Fluor 647	D57.2.2E	Cell Signaling Technology	4851	1:133 (FC)
SLP-76 (pY128)	Alexa Fluor 647	J141-668.36.58	BD Biosciences	558438	1:33 (FC)
STAT3 (pY705)	Alexa Fluor 647	4/P-STAT3	BD Biosciences	557815	1:10 (FC)
T-bet	PE	4B10	eBioscience	12-5825-82	1:50 (FC)
TNF-α	Brilliant Violet 421	Mab11	BioLegend	502932	1:50 (FC)
ZAP70 (pY319)	Alexa Fluor 647	17A/P-ZAP70	BD Biosciences	557817	1:10 (FC)
CD28	unconjugated	CD28.2	eBioscience	16-0289-85	1 µg/ml (S)
CD3	unconjugated	OKT3	eBioscience	16-0037-85	5 µg/ml (S)
CD25	Biotin	BC96	eBioscience	13-0259-82	1:10 (D)
CD69	Biotin	FN50	eBioscience	13-0699-82	1:10 (D)
HLA-DR	Biotin	LN3	eBioscience	13-9956-82	1:10 (D)
