# Supplementary material for: Sensing of HIV-1 by TLR8 activates human T cells and reverses latency
Source: Nat Commun. 2020 Jan 9;11:147. doi: 10.1038/s41467-019-13837-4 (PMC6952430; doi:10.1038/s41467-019-13837-4)
Supplement: Supplementary file 3 — Reporting Summary [file 41467_2019_13837_MOESM3_ESM.pdf]

nature research

Corresponding author(s): NCOMMS-18-34046B

Last updated by author(s): Nov 21, 2019

Reporting Summary

Nature Research wishes to improve the reproducibility of the work that we publish. This form provides structure for consistency and transparency in reporting. For further information on Nature Research policies, see Authors & Referees and the Editorial Policy Checklist .

Statistics

For all statistical analyses, confirm that the following items are present in the figure legend, table legend, main text, or Methods section.

n/a	Confirmed	
☐	☒	The exact sample size (*n*) for each experimental group/condition, given as a discrete number and unit of measurement
☐	☒	A statement on whether measurements were taken from distinct samples or whether the same sample was measured repeatedly
☐	☒	The statistical test(s) used AND whether they are one- or two-sided<br>*Only common tests should be described solely by name; describe more complex techniques in the Methods section.*
☐	☒	A description of all covariates tested
☐	☒	A description of any assumptions or corrections, such as tests of normality and adjustment for multiple comparisons
☐	☒	A full description of the statistical parameters including central tendency (e.g. means) or other basic estimates (e.g. regression coefficient) AND variation (e.g. standard deviation) or associated estimates of uncertainty (e.g. confidence intervals)
☐	☒	For null hypothesis testing, the test statistic (e.g. *F*, *t*, *r*) with confidence intervals, effect sizes, degrees of freedom and *P* value noted<br>*Give P values as exact values whenever suitable.*
☒	☐	For Bayesian analysis, information on the choice of priors and Markov chain Monte Carlo settings
☒	☐	For hierarchical and complex designs, identification of the appropriate level for tests and full reporting of outcomes
☒	☐	Estimates of effect sizes (e.g. Cohen's *d*, Pearson's *r*), indicating how they were calculated

Our web collection on statistics for biologists contains articles on many of the points above.

Software and code

Policy information about availability of computer code

Data collection	Flow-cytometry: FACS Diva Software, FlowSight Ideas Software, Confocal Microscopy: Las X, Zen, ELISA: BioRad Microplate Manager6, Multiplex ELISA: Bioplex Manager 6.1, Gel electrophoresis: GelEval v1.37, Western Blotting: Licor Studio software
Data analysis	GraphPad (Prism) V8<br>Flow cytometry data: FlowJo_V10, Cytobank, Amnis IDEAS<br>ELISA data: BioRad Microplate Manager6<br>Multiplex data: Bioplex Manager 6.1<br>Image analysis: Fiji Software

For manuscripts utilizing custom algorithms or software that are central to the research but not yet described in published literature, software must be made available to editors/reviewers. We strongly encourage code deposition in a community repository (e.g. GitHub). See the Nature Research guidelines for submitting code & software for further information.

Data

Policy information about availability of data

All manuscripts must include a data availability statement. This statement should provide the following information, where applicable:

- Accession codes, unique identifiers, or web links for publicly available datasets
- A list of figures that have associated raw data
- A description of any restrictions on data availability

All data that supports the plots within the manuscript and supplementary information are available from the corresponding author upon reasonable request. No restrictions on data availability. A list of figures that have associated raw data is included with reference to the Source Data file that is submitted with the manuscript.

Field-specific reporting

Please select the one below that is the best fit for your research. If you are not sure, read the appropriate sections before making your selection.

☒ Life sciences ☐ Behavioural & social sciences ☐ Ecological, evolutionary & environmental sciences

For a reference copy of the document with all sections, see nature.com/documents/nr-reporting-summary-flat.pdf

Life sciences study design

All studies must disclose on these points even when the disclosure is negative.

Sample size	Sample size did not need to be determined for this study.
Data exclusions	No data was excluded from the analysis.
Replication	All experiments were replicated as described in the figure legends
Randomization	Not needed for this study.
Blinding	N/A

Reporting for specific materials, systems and methods

We require information from authors about some types of materials, experimental systems and methods used in many studies. Here, indicate whether each material, system or method listed is relevant to your study. If you are not sure if a list item applies to your research, read the appropriate section before selecting a response.

Materials & experimental systems

n/a	Involved in the study	
☐	☒	Antibodies
☐	☒	Eukaryotic cell lines
☒	☐	Palaeontology
☒	☐	Animals and other organisms
☐	☒	Human research participants
☒	☐	Clinical data

Methods

n/a	Involved in the study	
☒	☐	ChIP-seq
☐	☒	Flow cytometry
☒	☐	MRI-based neuroimaging

Antibodies

Antibodies used

Information on all antibodies used in the study is provided in Supplementary Table 2.

eBioscience:
CD4 Alexa Fluor 700 (mIgG2b, clone OKT4, #56-0048-82)
CD14 PE/Cy7 (mIgG1, clone 61D3, #25-0149-42)
CD19 PE (mIgG1, clone HIB19, #12-0199-42)
CD11c PE (mIgG1, clone 3.9, #12-0116-42)
IFN-γ PE (mIgG1, clone 4S.B3, #12-7319-42)
IL-2 PE/Cy7 (mIgG2a, clone MQ1-17H12, #25-7029-42)
IkB alpha (pS32/pS36) eFluor 660 (mIgG2a, clone RILYB3R, #50-9035-42)
T-bet PE (mIgG1, clone 4B10, #12-5825-82)
RORγt APC (mIgG2a, clone AFKJS-9, #17-6988-82)
CD25 Biotin (mIgG1, clone BC96, #13-0259-82)
CD69 Biotin (mIgG1, clone FN50, #13-0699-82)
HLA-DR Biotin (mIgG2b, clone LN3, #13-9956-82)

BioLegend :
CD3 Brilliant Violet 785 (mIgG2a, clone OKT3, #317330)
CD8 Brilliant Violet 605 (mIgG2a, clone M5E2, #301814)
CD25 Brilliant Violet 510 (mIgG1, clone M-A251, #356120)
CD40L FITC (mIgG1, clone 24-31, #310804)
CD69 PE (mIgG1, clone FN50, #310906)

CD80 PE/Cy7 (mIgG1, clone 2D10, #305218)
HLA-DR Brilliant Violet 785 (mIgG2a, clone L243, #307642)
PD-1 Brilliant Violet 605 (mIgG1, clone EH12.2H7, #329924)
IL-17 Brilliant Violet 510 (mIgG1, clone BL168, #512330)
TNF-α Brilliant Violet 421 (mIgG1, clone Mab11, #502932)
CD45RO PerCP/Cy5.5 (mIgG2a, clone UCHL1, #304222)
CD45RA Brilliant Violet 785 (mIgG2b, clone HI-100, #304140)
CCR5 (CD195) PE/Cy7 (rat IgG2b, clone J418F1, #359107)
CXCR4 (CD184) PE/Cy5 (mIgG2a, clone 12G5, #306507)

BD Biosciences:
CD80 FITC (mIgG1, clone L307, #557226)
CD16 FITC (mIgG1, clone NKP15, #347523)
CD3ζ (pY142) Alexa Fluor 647 (mIgG2a, clone K25-407.69, #558489)
ZAP70 (pY319) Alexa Fluor 647 (mIgG1, clone 17A/P-ZAP70, #557817)
SLP-76 (pY128) Alexa Fluor 647 (mIgG1, clone J141-668.36.58, #558438)
NF-κB p65 (pS529) Alexa Fluor 647 (mIgG2b, K10-895.12.50, #558422)
STAT3 (pY705) Alexa Fluor 647 (mIgG2a, clone 4/P-STAT3, #557815)

Miltenyi Biotec:
CD303 APC (mIgG1, clone AC144, #130-113-190)
CD123 PE (mIgG2a, clone AC145, #130-113-326)
CD25 PE (mIgG2b, clone 4E3, #130-113-282)
IL-4 PE-Vio615 (mIgG1, clone 7A3-3, #130-107-144)
IFN-γ FITC (rec. human IgG1, REA600, #130-113-497)

Cell Signaling Technology
TLR8 (rabbit mAb, clone D3Z6J, #11886)
Akt (pS473) Alexa Fluor 647 (rabbit mAb, clone D9E, #4075)
S6 Ribosomal Protein (pS235/pS236) Alexa Fluor 647, (rabbit mAb, clone D57.2.2E, #4851)
NF-κB p65 (pS536) Alexa Fluor 647 (rabbit mAb, clone 93H1, #4887)
p44/42 MAPK (Erk1/2, pT202/pY204) Alexa Fluor 647 (rabbit mAb, clone E10, #4375)
p38 MAPK (pT180/pY182) Alexa Fluor 647 (mIgG1, clone 28B10, #4552)

Santa Cruz
LAMP1 (mIgG1, clone H4A3, #sc-20011)
EEA1 (rabbit polyclonal, Santa Cruz H-300, #sc-33585)

Abcam
LAMP1 (rabbit polyclonal, #ab24170)
HIV1 p24 (mIgG1, clone 39/5.4A, #ab9071)

Validation

All antibodies are commercially available and were validated by the supplier.

Eukaryotic cell lines

Policy information about cell lines

Cell line source(s)

Cell lines were purchased from ATCC.

Authentication

No further authentication procedures were performed.

Mycoplasma contamination

Cell lines are routinely tested in our facilities and tested negative for mycoplasma.

Commonly misidentified lines
(See ICLAC register)

No commonly misidentified cell lines were used.

Human research participants

Policy information about studies involving human research participants

Population characteristics	The study was performed on blood cells from healthy donors and HIV- infected patients. Blood cells from healthy donors were isolated from buffy coats provided by the Blood Bank (St Olavs Hospital, Trondheim). No additional information (age, sex) was given for these donors.<br>The study population of HIV-1 infected patients included blood samples from 9 individuals aged 27 - 69 years old and included 2 women and 7 men. None of the patients had other chronic inflammatory diseases or used medication affecting inflammation. Additional details on HIV medication and viral load are given in Supplementary Table 1.
Recruitment	Buffy coats for blood cell isolation from healthy blood donors were provided by the Blood Bank (St Olavs Hospital, Trondheim). Blood samples from HIV-1 infected patients were recruited from the St. Olav's infectious disease out-patient clinic. The participants were on ART and had suppression of plasma HIV-1 viremia. All participants provided written informed consent to participate in the study.
Ethics oversight	This project was approved by the Regional Committee for Medical and Health Research Ethics (REC Central, Norway) for blood samples from healthy donors (NO. 2009/2245) and HIV-1 infected patients (REK 2014/1507). REC Central Norway is a committee equivalent to Institutional Review Board in USA.

Note that full information on the approval of the study protocol must also be provided in the manuscript.

Flow Cytometry

Plots

Confirm that:

- [x] The axis labels state the marker and fluorochrome used (e.g. CD4-FITC).
- [x] The axis scales are clearly visible. Include numbers along axes only for bottom left plot of group (a 'group' is an analysis of identical markers).
- [x] All plots are contour plots with outliers or pseudocolor plots.
- [x] A numerical value for number of cells or percentage (with statistics) is provided.

Methodology

Sample preparation	CD4+ T cells were isolated and prepared as described in the Methods section.
Instrument	BD LSR II, BD LSR Fortessa, Amnis FlowSight
Software	FACS Diva software for acquisition, FlowJo_V10 and Cytobank software for analysis, Amnis IDEAS software
Cell population abundance	Purity was in excess of 94% for each CD4+ T cell subset MACS isolation
Gating strategy	Lymphocytes: positively identified from FSC/SSC, single cells: positively identified from FSC-A/FSC-H, viable cells: negative fraction from FSC/viability dye, CD4+ T cells: Identified as CD3+/CD4+ fraction of viable lymphocytes or as cell tracker positive lymphocytes in HIV-1 infection experiments. Further parameters such as cytokine-production, activation marker and transcription factor expression, memory/naive T cell subsets and HIV-1 infection were identified as described in the manuscript. Gating for these parameters was performed on unstimulated or uninfected cells. The different gating strategies are exemplified in Supplementary Figure S7.

- [x] Tick this box to confirm that a figure exemplifying the gating strategy is provided in the Supplementary Information.
